# Meta-analysis of gut microbiome reveals patterns of dysbiosis in colorectal cancer patients

**DOI:** 10.1099/jmm.0.002042

**Published:** 2025-07-30

**Authors:** Ranxin Yan, Rui Zheng, Yucheng Han, Ge Song, Ban Huo, Han Sun

**Affiliations:** 1 College of Economics and Management, Henan Agricultural University, Zhengzhou 450046, PR China; 2 College of Information and Management Science, Henan Agricultural University, Zhengzhou 450046, PR China; 3 College of Veterinary Medicine, Henan Agricultural University, Zhengzhou 450046, PR China; 4 School of Mathematics and Computer Science, Hanjiang Normal University, Shiyan 442000, PR China

**Keywords:** batch effect, colorectal cancer, differential abundance analysis, gut microbiome, microbiome-based association test

## Abstract

**Introduction.** Colorectal cancer (CRC) is a malignant tumour in which dysbiosis of the gut microbiome is a contributing factor in the development of cancer. However, the species composition and species-specific changes in the gut microbiome related to CRC still require comprehensive investigation.

**Hypothesis.** There is a significant difference in gut microbiome between CRC patients and healthy individuals.

**Aim.** The microbiome-based association test methods are used for the association between the microbiome and host phenotypes, and linear discriminant analysis effect size (LEfSe) analysis is employed to search for microbial biomarkers associated with CRC.

**Methodology.** We conducted a meta-analysis of microbiome data from multiple cohorts, totalling 1,462 samples and 320 genus-level features. Considering the data obtained under different experimental conditions, we removed the batch effect using conditional quantile regression. Then, we employed the common analysis processes and methods of microbiome data, including microbial diversity analysis, microbiome-based association test analysis and microbial differential abundance analysis.

**Results.** The experimental results showed that there were significant differences in *α*-diversity between the CRC group and the healthy group, as well as in the overall microbial community (PERMANOVA *P*-value less than 0.05). LEfSe analysis also demonstrated the genus-level features enriched in the gut of CRC patients and the genus-level features enriched in the gut of healthy individuals. Notably, the batch effect-corrected data exhibit more significant performance than the raw data.

**Conclusion.** Gut microbiome composition is a significant factor associated with the development of CRC. *Enterobacter* and *Fusobacterium* enriched in the gut of CRC patients may be CRC-related microbial biomarkers, while *Bacteroides* and *Faecalibacterium* enriched in the gut of healthy individuals are core genera of the healthy gut. In addition, batch effects in microbiome data caused by differences in sample handling may lead to false discoveries, especially large-scale microbiome data. These findings could deepen the understanding of the role played by gut microbes in CRC and are expected to provide recommendations for the diagnosis of cancer and the development of new microbial therapies.

## Data Summary

Requests for sharing further information, including data and code, for scientific research can be directed to the corresponding author.

## Introduction

In recent years, the number of people suffering from colorectal cancer (CRC) has been steadily increasing worldwide, making it the fourth most deadly cancer globally [1,2]. Currently, the incidence of CRC has stabilized, and mortality is declining in highly developed countries [3]. However, the incidence of CRC in developing countries is rapidly rising due to an increase in CRC risk factors and the lack of large-scale screening programmes [4]. Early-stage CRC is often asymptomatic, and it is usually at an advanced stage by the time it is detected, making treatment more difficult. To improve CRC survival rates, the identification of biomarkers is crucial for screening, early diagnosis, prognosis and understanding the pathogenesis of CRC [5]. The Human Microbiome Project (HMP) [6] has revealed mechanisms by which microbes influence host phenotypes, including disease [7,8]. The gut microbial community is closely linked to the body’s immune system and metabolism, and disruptions in this community can lead to a range of health problems, including CRC [9]. Numerous studies have demonstrated that the gut microbiome plays a significant role in the development of CRC, influencing carcinogenesis, tumour formation and progression, as well as responses to various systemic therapies [10]. Therefore, studying the gut microbiome in CRC patients is essential.

The relationship between CRC and gut microbiome has always been one of the hot topics in microbiome studies. A growing number of studies have shown that CRC progression is associated with gut microbiome composition. Researchers have identified CRC-specific global microbial profiles through meta-analysis of faecal metagenomes [11]. This meta-analysis firmly establishes globally applicable, predictive taxonomic and functional microbiome CRC profiles as a basis for future diagnostics. In addition, the advances in CRC microbiome studies need to consider how the microbiome promotes CRC carcinogenesis through different pathways that lead to CRC, thereby creating new prevention, screening and treatment interventions [12]. Researchers have discussed the role of gut microbes in the growth and progression of CRC and their synergistic therapeutic modalities with anticancer treatments. They describe the strains of the gut microbiome that influence each stage of the tumourigenesis process [13], providing an overview of the possible involvement of gut microbiome species in future studies on the relationship between the gut microbiome and CRC.

In recent years, there has been increasing evidence that specific microbial species can play a key role in the progression of CRC through multiple mechanisms. For example, a 2024 study found that the *Fusobacterium nucleatum* causative agent *Porphyromonas gingivalis* exacerbates intestinal inflammation in a gut microbial-dependent manner. The mechanism is to induce a decrease in the levels of metabolic product linoleic acid by affecting the structure of the gut microbiota, which in turn promotes an imbalance between inflammatory Th17 cells and immunosuppressive Treg cells, ultimately aggravating colitis, which may lead to the development of CRC [14]. In addition, recent studies have shown that *F. nucleatum* is believed to produce genetically toxic compounds that are capable of causing DNA damage in host cells, thereby promoting the progression of genetic mutations and CRC [15]. For example, metabolic products of certain bacteria can cause DNA damage in intestinal epithelial cells and induce non-dependent apoptosis of p53 [16,18]. Understanding these microbial-mediated mechanisms is critical to developing targeted therapeutic strategies for CRC. However, microbiome studies on CRC often face challenges due to insufficient sample sizes. The studies that collect microbiome data from different cohorts of CRC patients and healthy individuals through meta-analyses hardly consider the batch effects in microbiome data processing.

To address the influence of batch effects, batch effect correction methods based on partial least squares discriminant analysis (PLSDA-batch) are proposed [19], which is a new correction method for multivariate and nonparametric batch effects based on PLSDA. However, a limitation of this method is that it requires microbial relative abundance data for correction, and the corrected data remain as microbial relative abundance. Considering the conversion relationship between relative and absolute abundance data, it may limit our subsequent analysis. A conditional quantile regression (ConQuR) method [20] uses a two-part quantile regression model to remove the batch effect of the microbiome. By using absolute abundance modelling, this method effectively avoids the limitations of the previous method for this study. In addition, other methods for dealing with batch effects are being developed. The MMUPHin method [21] is a computational framework for meta-analysis of microbiome data that enables batch effect correction while integrating multiple datasets to identify robust microbial associations. An improved batch effect estimation method [22] is introduced based on deep learning with non-linear fitting.

**Fig. 1.**
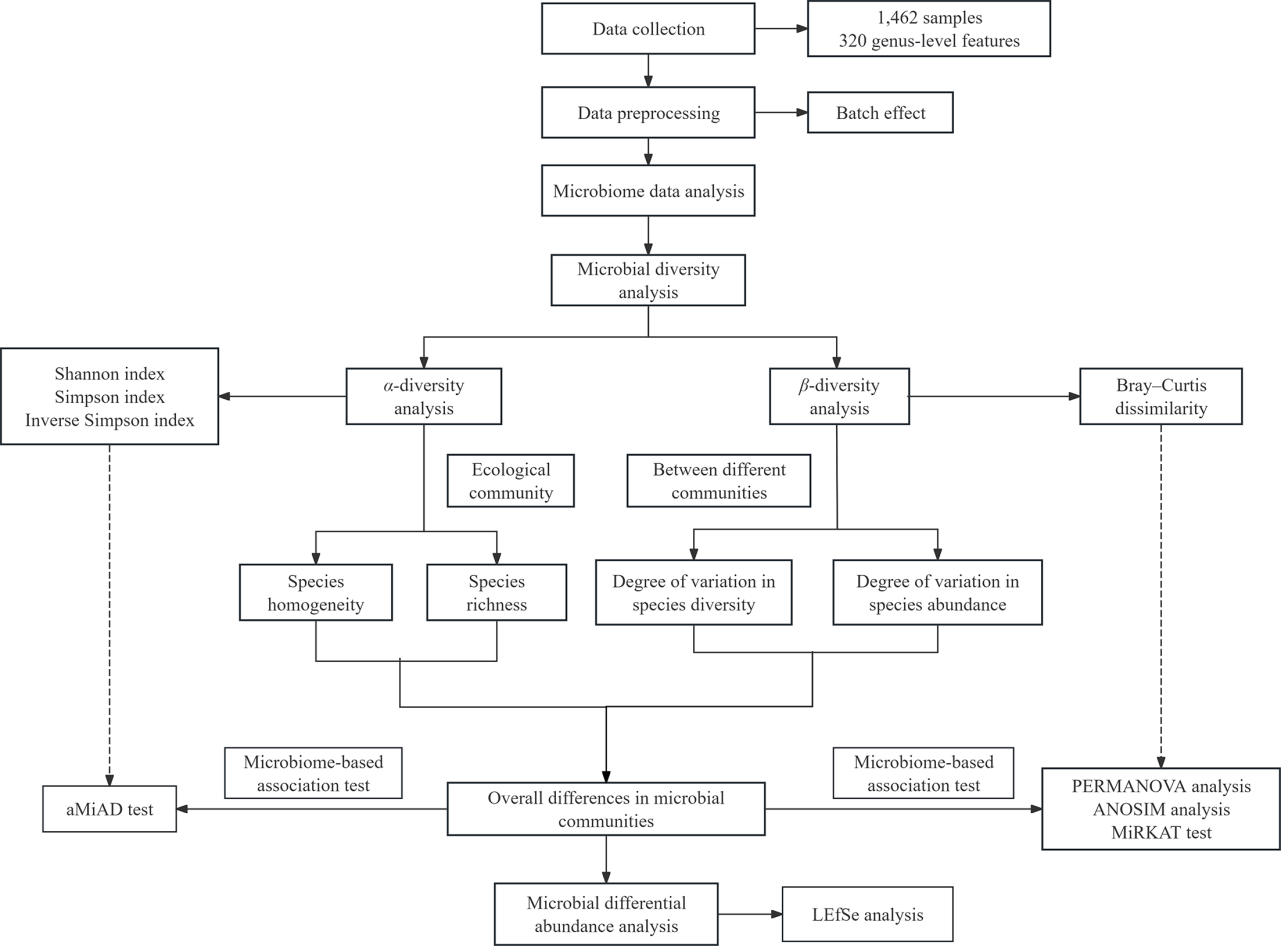
Schematic diagram of overall experimental analysis. We collected a dataset containing 1,462 samples with 320 genus-level features, and we used a ConQuR method for data preprocessing. We then conducted the microbial diversity analysis to investigate the diversity of gut microbiome in CRC patients and healthy individuals. In the *α*-diversity analysis, we employed the Shannon index, Simpson index and inverse Simpson index to assess species within the community, identifying species evenness and species richness. We also utilized the Bray–Curtis dissimilarity for *β*-diversity analysis between different communities, exploring the differences in species diversity and species richness. Meanwhile, we applied the microbiome-based association test analysis, including aMiAD method, PERMANOVA analysis, ANOSIM analysis and MiRKAT method, to test the association between the gut microbiome and CRC. In addition, we performed the microbial differential abundance analysis using LEfSe analysis to identify biomarkers associated with CRC.

We first collected six batches of microbiome data based on a previous study [23], and by combining data from different batches, we obtained a dataset containing 1,462 samples with 320 genus-level features. The large-scale microbiome data can enhance the statistical significance and reliability of the results. Given that the dataset is from various studies, the batch effect of microbiome data can significantly impact downstream analyses. Therefore, we adopted ConQuR to remove microbiome batch effects and validated the improved results of corrected data through extensive experiments. Then, comprehensive microbiome data analyses were performed, including microbial diversity analysis, microbiome-based association tests and microbial differential abundance analysis (Fig. 1). Specifically, we used the Shannon index, Simpson index and the inverse Simpson index for *α*-diversity analysis to assess species evenness and richness within the samples. Adaptive microbiome *α*-diversity-based association analysis (aMiAD) [24] was also used to verify the differences in *α*-diversity between CRC patients and healthy individuals. In addition, we used the Bray–Curtis dissimilarity [25] to analyse the *β*-diversity of species among different communities, exploring the differences in species diversity and richness between CRC patients and healthy individuals. Permutational multivariate analysis of variance (PERMANOVA) [26], analysis of similarity (ANOSIM) [27] and microbiome regression-based kernel association test (MiRKAT) [28] were used to verify the overall differences in microbial communities between CRC patients and healthy individuals. Finally, we performed microbial differential abundance analysis using the linear discriminant analysis (LDA) effect size (LEfSe) method [29] to identify microbial biomarkers related to CRC through the taxonomic branching diagram and histograms of LDA value distribution. We found that the gut microbiome of CRC patients was significantly enriched in *Enterobacter* and *Fusobacterium*, which may contribute to the diagnosis, prediction or treatment of CRC.

## Methods

### Data collection

In this study, we systematically collected and analysed gut microbiome data from CRC patients and healthy individuals. The inclusion criteria for selecting datasets were based on the availability of 16S rRNA gene sequencing data, human subjects, annotation information at the genus level and a clear distinction between CRC patients and healthy controls. Studies lacking sufficient metadata, with data quality issues or being duplicates/overlapping were excluded. Detailed data collection and preprocessing steps are described in the following sections.

We used the dataset based on a previous study [23], which collected gut microbiome data from the National Center for Biotechnology Information (NCBI) for a wide range of disease populations. Keywords searched in PubMed included ‘gut microbiota’, ‘16S’, ‘human’, ‘stool’ and ‘microbiome’. A total of 5,608 faecal samples from diseased individuals and 5,769 healthy control samples from 78 studies were collected. The inclusion criteria for selecting datasets were as follows: (1) Studies must have included 16S rRNA gene sequencing data for the gut microbiome. (2) Samples must have been collected from human subjects. (3) Studies must have provided annotation information at the genus level. (4) Studies must have clearly distinguished between CRC patients and healthy controls. The exclusion criteria were as follows: (1) Studies lacking sufficient metadata for sample annotation. (2) Studies with data quality issues, such as high levels of contamination or low sequencing depth. (3) Duplicate studies or studies with overlapping sample sets. The raw 16S rRNA gene sequencing data were constructed into an amplicon sequence variant (ASV) feature table by using the bioinformatic tools QIIME2 [30] and DADA2 [31], and ASVs with relative abundance above 0.1% were considered for inclusion in the experiment. For this study, we specifically selected some samples related to CRC. The data we used in our experiment consisted of 6 batches, each further divided into healthy samples and CRC samples, resulting in 12 datasets. Each dataset included a varying number of samples and features, with the features being ASVs specific to the genus level. We constructed our dataset by combining these 12 datasets and retaining microbiome common to both CRC and healthy samples. Microbiome data are usually high-dimensional, sparse and compositional. Our dataset contains a total of 1,462 samples and 320 features, including 674 healthy samples and 788 CRC samples. During the data collection process, we found that these data only have annotation information at the genus level (e.g. g_*Pseudomonas*), without more complete annotation information such as phylum and family levels (e.g. d_*Bacteria*, p_*Proteobacteria*, c_*Gammaproteobacteria*, o_*Pseudomonadales* and f_*Pseudomonadaceae*). To ensure data completeness and downstream analysis, we supplemented each annotation and sequence information sequentially through the NCBI.

### Data preprocessing

Although large-scale studies can provide more significant and reliable analysis, they can also be severely affected by batch effects [20], especially multiple cohorts, due to differences in time, operators, reagents and instruments. Therefore, when conducting a meta-analysis on the fusion of microbiome data from different batches, we need to consider the impact of these batch effects. Batch effect processing involves isolating batch information that may affect the signal of key variables to generate batch-free data suitable for subsequent analyses. Batch effect processing adjustments, on the other hand, label batch information as a covariate to enable all commonly used microbiome analyses [19]. In recent years, many approaches to deal with batch effects have been proposed [19,22]. Considering the requirements of our data analysis, we adopted ConQuR [20], a comprehensive microbiome batch effect removal tool. ConQuR starts by assuming the probability of observing non-zero features and identifies the effect of batch information by automatically capturing the variations between batches. It then eliminates the effect of batch information by setting parameters to ignore the batch effect. ConQuR requires specifying the reference batch data. We analysed different batches as reference batches and ultimately adopted the third batch data as the reference batch data. Detailed comparison results can be found in the ‘Composition and preprocessing of microbiome data’ section.

### Microbial diversity analysis

We analysed the *α*-diversity and *β*-diversity of CRC patients and healthy individuals from microbiome data. Given the lack of phylogenetic information, we used only common diversity indices without phylogenetic information. Here, *α*-diversity mainly focuses on the diversity within a specific region or ecosystem, and *α*-diversity indices we used included the Shannon index, Simpson index and inverse Simpson index. Specifically, the Shannon index considers species richness and evenness, calculating diversity based on the relative abundance of individual species in the sample. The higher Shannon index value indicates greater species richness and more even distribution within the sample. The Simpson index value evaluates species richness in the sample, where the lower Simpson index value indicates greater species richness. The higher Simpson index value signifies a more heterogeneous community, with a few species dominating the community. The inverse Simpson index reflects the degree of evenness in the number of species in a community. Unlike the Simpson index, the higher the inverse Simpson index value, the more evenly distributed the number of species in the community, indicating higher diversity. We used the *diversity* function in the vegan R package [32] to calculate these three *α*-diversity indices. In addition, *β*-diversity describes the differences in species composition or abundance between samples, recording the species that occur in different samples and their relative abundance. We used the Bray–Curtis dissimilarity [25], a non-phylogeny-based distance metric, for *β*-diversity calculations. The large Bray–Curtis dissimilarity value indicates the differences between two samples including composition and abundance. We used the *bcdist* function from the ecodist R package [33] to calculate the Bray–Curtis dissimilarity.

### Microbiome-based association test analysis

To further validate the overall differences in microbial communities between CRC patients and healthy individuals, we employed many microbiome-based association tests, including PERMANOVA analysis [26], ANOSIM analysis [27], aMiAD method [24] and MiRKAT method [28]. Here, PERMANOVA, ANOSIM and MiRKAT are classic distance-based test methods that can be used to test the global effect in microbial communities [34], and aMiAD is an adaptive test method for evaluating the differences in *α*-diversity of microbial communities in different groups. Specifically, PERMANOVA uses a distance matrix to decompose the total variance and analyse the degree of explanation of sample differences by different grouping factors. ANOSIM is mainly used to analyse the similarity between groups of high-dimensional data, providing a basis for evaluating the significance of differences between the data. We performed PERMANOVA and ANOSIM analyses using the *adonis2* and *anosim* functions in the vegan R package, respectively. Both aMiAD and MiRKAT use generalized linear models to construct the relationship among host phenotype, confounding factors and microbiome, which allows them to adjust for confounding factors while testing the association between the microbiome and host phenotype. Unfortunately, the missing information resulted in the failure to further adjust for confounding factors. In addition, the aMiAD method and MiRKAT method construct test statistics based on *α*- and *β*-diversity information, respectively, and the aMiAD method uses the minimum *P*-value method [35] to fuse the individual tests based on different diversity indices. All of these tests use permutations to calculate *P*-values.

### Microbial differential abundance analysis

Microbial differential abundance analysis is an essential topic in microbiome data analysis [36], which can be used to identify disease-related microbial biomarkers. We employed a classic and popular method (LEfSe analysis) [29,37] for identifying differentially abundant taxa. LEfSe analysis first used the non-parametric factorial Kruskal–Wallis sum-rank test [38] to test the significant differences in abundance between different groups. Then, for the significant difference features obtained, the pairwise Wilcoxon rank-sum test [39] was performed for the subgroups of different groups to further analyse the differences between groups. Finally, the LDA was used to evaluate the influence of species with significant differences, where the ranking of biomarker relevance could be obtained through LDA score. Considering the potential for false discoveries, we employ the Bonferroni correction [40] to adjust *P*-values for multiple hypothesis testing. Specifically, we set the threshold value for *P*-value of the Kruskal–Wallis sum-rank test and LDA score to 0.05/*M* and 2.5, respectively, where *M* represents the number of features.

## Result

### Composition and preprocessing of microbiome data

**Fig. 2.**
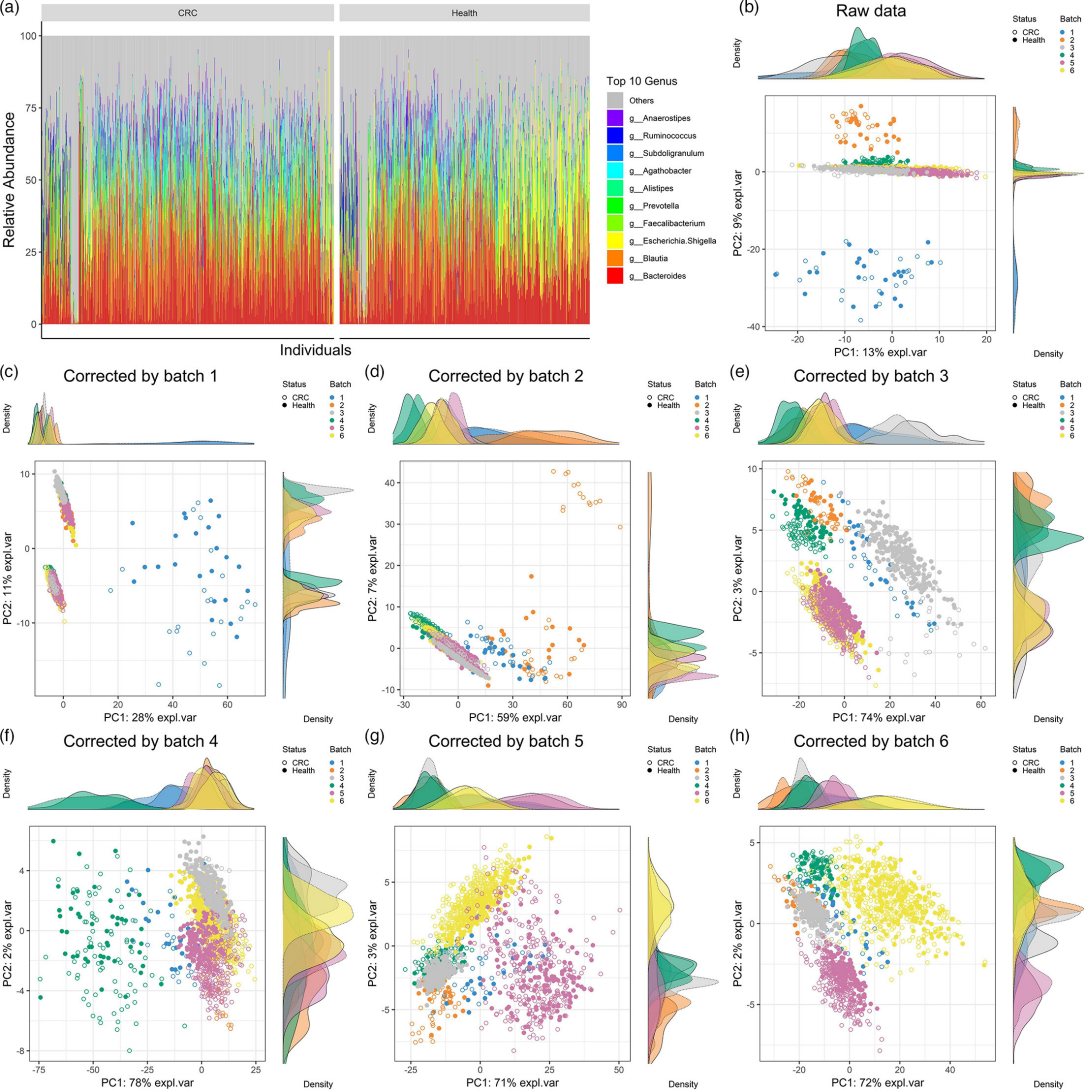
Composition of gut microbial communities and batch effect processing of microbiome data. (**a**) The top ten genus-level features with the highest relative abundance. Principal component analysis plots for the raw data (**b**) and corrected data (**c–h**) using batches 1–6 as reference batches, respectively.

The microbiome data we used contained 1,462 samples with 320 features. We converted the microbiome abundance data into relative abundance data and found that the top ten ASVs in terms of average relative abundance were *Bacteroides*, *Blautia*, *Escherichia-Shigella*, *Faecalibacterium*, *Prevotella*, *Alistipes*, *Agathobacter*, *Subdoligranulum*, *Ruminococcus* and *Anaerostipes*, respectively (Fig. 2a). Here, ‘other’ represents the rest of ASVs. *Bacteroides* is a crucial cornerstone genus in humans, widely present in the human gut, and it maintains a symbiotic relationship with the host [41]. *Blautia*, *Escherichia-Shigella* and other genera are also core components of the gut microbiome.

We compared the principal component analysis (PCA) plots before and after batch effect correction to evaluate the performance of the raw and corrected data [20]. Considering that the relative abundance data were compositional and belonged to the simplex space rather than the common Euclidean space, we transformed the data using centred log-ratio (CLR). Then, we employed PCA to analyse the transformed data. The raw and corrected microbial relative abundance data were analysed by using CLR transformation and PCA, as shown in Fig. 2(b–h). For the PCA of the raw data (Fig. 2b), we could find that PC1 explains only 28% of the variance. The dispersion of distribution between different batches indicates a significant difference between batches. We adjusted the raw data by using the ConQuR method to remove microbiome batch effects. ConQuR requires specifying the reference batch data. We selected six batches of data one by one as reference batch data to adjust the raw data and compared the PCA plots (Fig. 2b). For the corrected data using Batch 1 and Batch 2 as the reference batch, we could find that PC1 still explains a limited amount of information and the batch data remain discrete (Fig. 2c, d). For the corrected data using Batch 3 through Batch 6 as the reference batch, we could find that PC1 explains more than 70% of the variance, and the data distribution between different batches is mostly intersecting (Fig. 2e–h), especially Batch 3. Considering the data distribution and the variance explained by the PC1 and PC2 axes, we adopted Batch 3 as the reference batch in the ConQuR method to adjust the raw data for subsequent analysis.

### Association between the microbiome and CRC

**Fig. 3.**
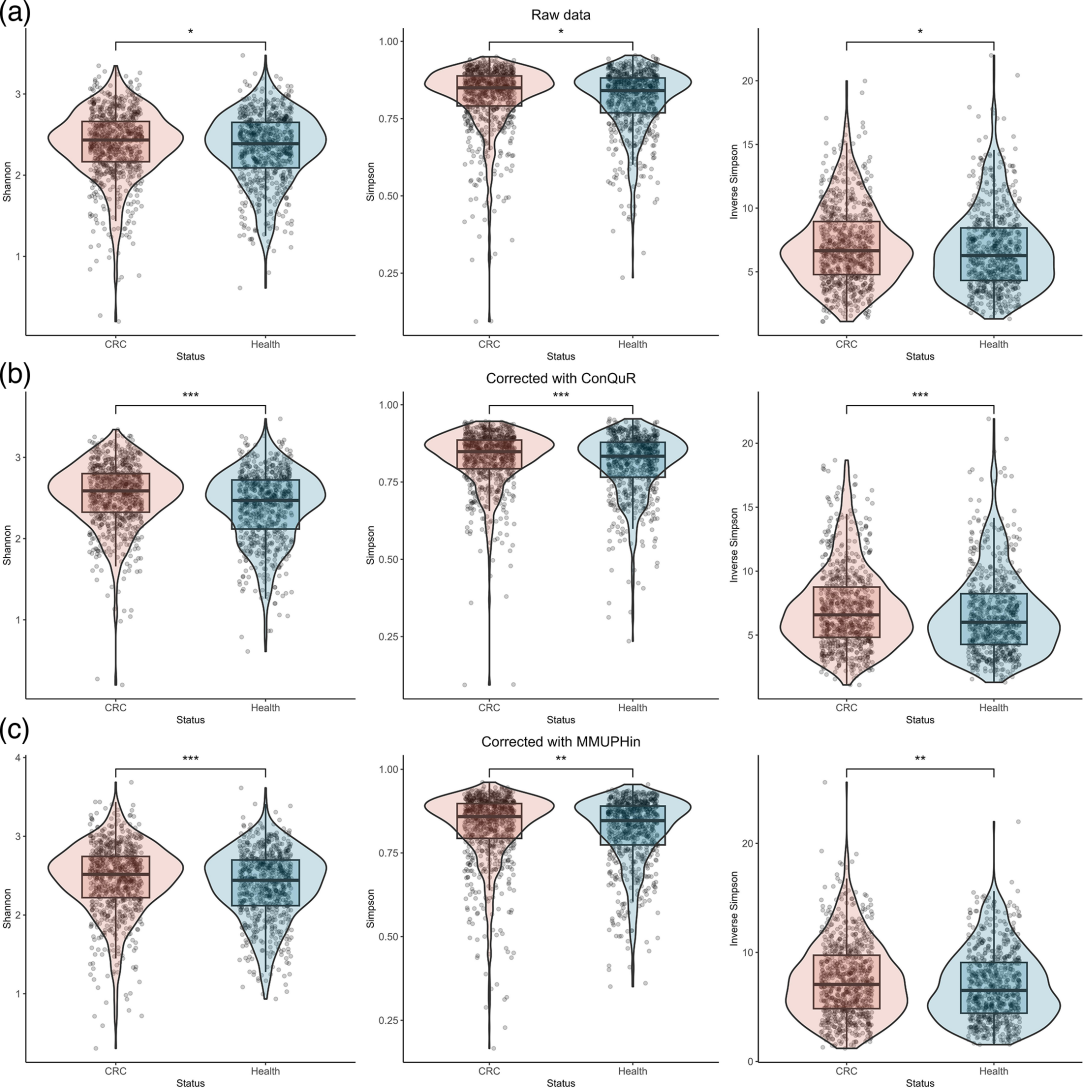
The diversity of the gut microbiome in CRC patients and healthy individuals, including the Shannon, Simpson and inverse Simpson indices. (**a**) Raw data analysis. (**b**) Corrected data with ConQuR. (**c**) Corrected data analysis with MMUPHin.

We analysed the *α*-diversity of gut microbiome in CRC patients and healthy individuals according to both raw and corrected data, respectively, including the Shannon index, Simpson index and inverse Simpson index (Fig. 3). A large portion of the Shannon index, Simpson index and inverse Simpson index values are between 2 and 3, 0.75 and 1 and 5 and 10, respectively. The Wilcoxon rank-sum test was performed on the *α*-diversity of the CRC and healthy samples. We could find that there are significant differences in the Shannon, Simpson and inverse Simpson indices between CRC and healthy samples, both in the raw data and the corrected data. Notably, the diversity differences in the corrected data are even more significant (i.e. ***) compared with the raw data (i.e. *) (Fig. 3). In addition, we also employed the aMiAD method to further verify the *α*-diversity differences between the raw and corrected data, including individual tests based on single diversity and adaptive tests (Table 1). For raw data, only individual test with the Shannon index can detect significant differences, while other test methods, including individual tests with two other *α*-diversity indices and adaptive test that integrates all three *α*-diversity indices, cannot detect differences. Conversely, for the corrected data, all tests used showed significant differences, further validating the effectiveness of the methodology used in this study to address batch effects.

**Table 1.** The results of aMiAD individual tests and adaptive test on raw data and corrected data

Diversity index	Raw data	Corrected data with ConQuR	Corrected data analysis with MMUPHin
Shannon	0.032*	<0.001*	0.001*
Simpson	0.094	<0.001*	0.072
Inverse Simpson	0.072	0.005*	0.004*
Adaptive test	0.057	<0.001*	0.003*

*P-values less than 0.05.

**Fig. 4.**
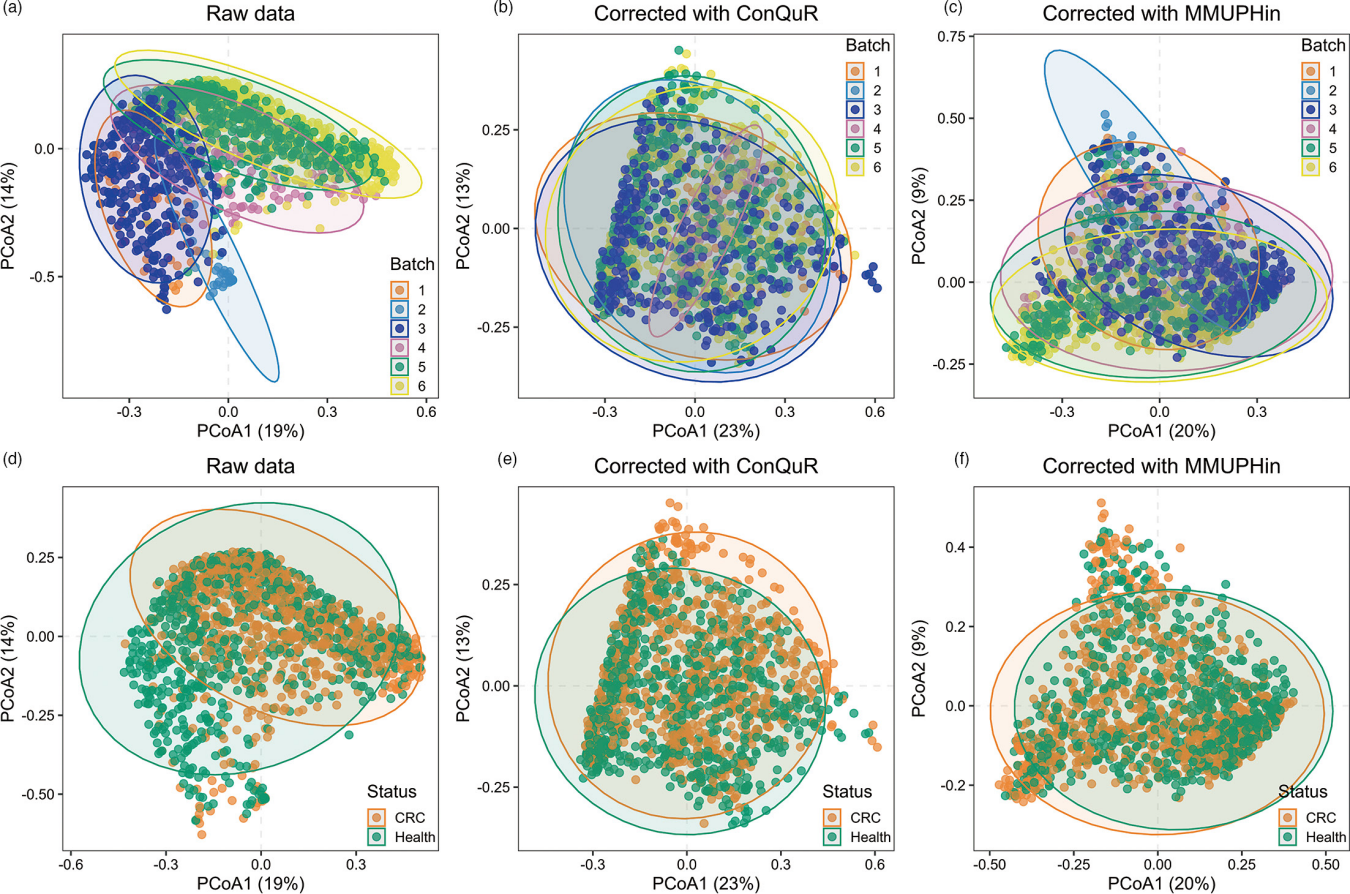
PCoA plot for the raw data and corrected data. (**a, d**) The raw data grouped by batch information and label information. (**b, e**) The corrected data with ConQuR grouped by batch information and label information. (**c, f**) The corrected data with MMUPHin grouped by batch information and label information.

We also conducted the principal coordinate analysis (PCoA) of the raw and corrected data based on the Bray–Curtis dissimilarity (Fig. 4). Fig. 4(a, d) shows the raw data grouped by batch information and label information, respectively, while Fig. 4(b, c, e, f) displays the corrected data grouped by batch information and label information, respectively. It is evident that the differences in the data caused by batch effects are reduced after adjustment by comparing Fig. 4(a, b). Meanwhile, we also found a certain degree of difference in microbial communities between CRC samples and healthy samples (Fig. 4d, e, f). To further verify this difference, we used PERMANOVA, ANOSIM and MiRKAT to analyse the raw and corrected data, respectively (Table 2). We found that the results before and after adjustment were basically the same, indicating significant differences in microbial communities between CRC samples and healthy samples (i.e. *P*-value less than 0.05).

**Table 2.** The results of distance-based tests on raw data and corrected data

Method	Raw data	Corrected data with ConQuR	Corrected data analysis with MMUPHin
PERMANOVA	0.001*	0.001*	0.001*
ANOSIM	0.001*	0.001*	0.003*
MiRKAT	<0.001*	<0.001*	<0.001*

*P-values less than 0.05.

Here, the distance metric used is the Bray–Curtis dissimilarity.

### Identification of microbial biomarkers

**Fig. 5.**
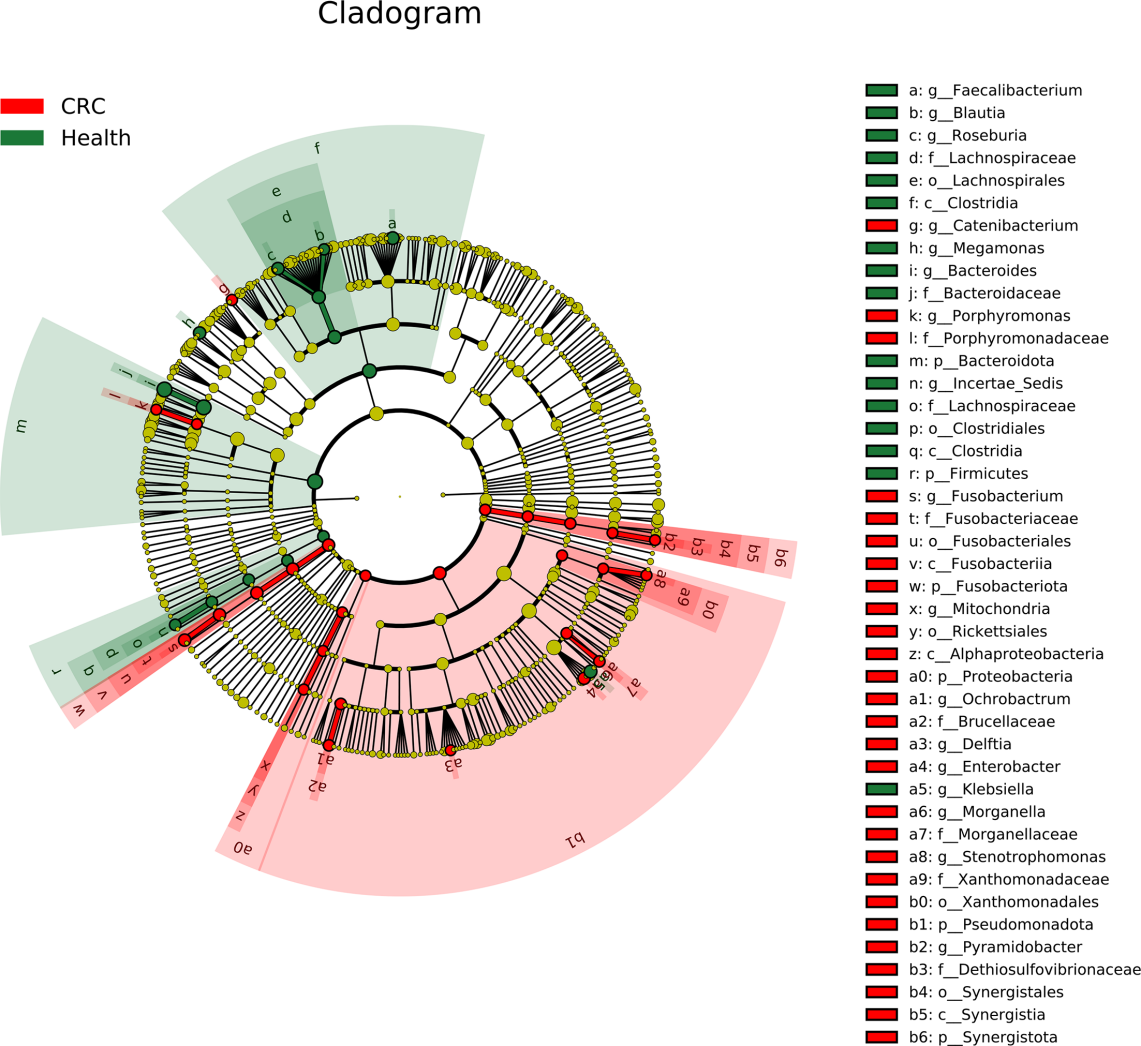
Taxonomic branching diagram of enriched biomarkers for LEfSe analysis of differences in gut microbiota composition between CRC patients and healthy individuals.

**Fig. 6.**
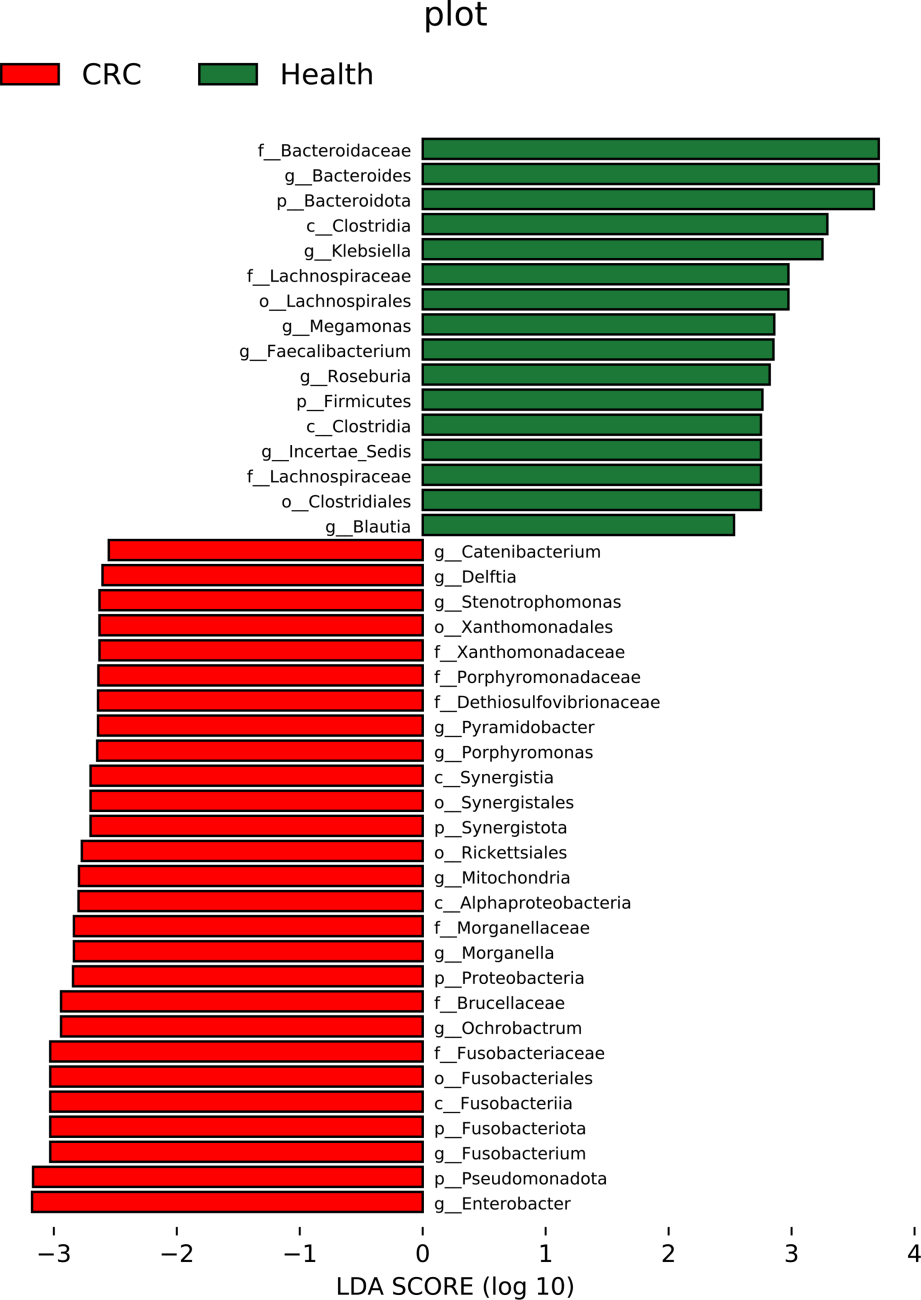
LDA value distribution histogram of the differential microbial community.

To identify biomarkers associated with CRC, we used LEfSe analysis for microbial differential abundance analysis. We completed the taxonomic branching diagram and histograms of LDA value distribution for enriched biomarkers (Figs5  6). For the taxonomic branching diagram, yellow nodes indicate ASVs that are not significantly different between CRC patients and healthy individuals, while other nodes represent ASVs with significant differences. Node size correlates with the abundance of ASVs, that is, the larger the node, the higher its abundance. The size of nodes is related to the abundance of ASVs, meaning that the larger the node, the higher the number or proportion of ASVs in the sample. We can identify significant differential features at various levels related to CRC/health as depicted in the taxonomic branching diagram (Fig. 5) and histograms of LDA value distribution (Fig. 6). Specifically, compared with the healthy group, some genus-level features in the CRC group have higher abundance, such as *Enterobacter* and *Fusobacterium*. It indicates that these bacteria are enriched in the gut of CRC patients, which have also been reported to be associated with CRC [15,42]. Specifically, *Enterobacter* is a strong 4-hydroxyphenylacetate producer.

In the healthy group, the abundance of some genus-level features, such as *Bacteroides*, *Klebsiella*, *Megamonas* and *Faecalibacterium*, is relatively higher. Here, *Bacteroides* and *Faecalibacterium* are core genera in a healthy gut, known for maintaining intestinal barrier function and regulating the immune system. When using raw data for LEfSe analysis, more bacteria with significant differences were obtained due to pseudo-associations caused by data differences between batches. Therefore, we only use corrected data for LEfSe analysis to identify microbial biomarkers.

## Discussion

In this article, we systematically investigated the characteristics of CRC patients’ gut microbiome and observed substantial changes in their gut microbiome compared with healthy individuals. Specifically, we merged multiple cohorts of CRC patients and healthy individuals to construct the dataset used in our experiment, and we preprocessed data from different batches using ConQuR to eliminate the influence of batch effects. The corrected data were then subjected to comprehensive microbiome data analyses, including microbial diversity analysis, microbiome-based association test analysis and microbial differential abundance analysis. Massive experimental analysis revealed the differences in *α*-diversity between the CRC group and the healthy group, as well as microbial community composition. Meanwhile, many microbiome-based association test methods validated these significant differences. In addition, microbial differential abundance analysis provided different enriched features in each group. The guts of CRC patients were significantly enriched with *Enterobacter* and *Fusobacterium*, which may provide new insights into the role of gut microbiome in the development of CRC [43,44]. It is crucial to understand the association between CRC and gut microbiome for identifying potential therapeutic targets and developing treatment strategies for related diseases. Our research provides specific microbial biomarkers (*Enterobacter* and *Fusobacterium*) that are significantly enriched in the gut of CRC patients. These biomarkers have the potential to enhance early CRC diagnosis through screening tests and may guide the development of targeted microbial therapies aimed at restoring the healthy gut microbiome, thereby contributing to more effective CRC treatment strategies.

Our current research reveals the association between gut microbiome and CRC, which helps to understand the role of the microbiome in the development of CRC and translate it into clinical practice. However, our findings may lack generalizability to populations of diverse geographic and ethnic backgrounds. The datasets we used in the meta-analysis were collected primarily from studies conducted in specific regions, and most of the samples were likely from relatively homogeneous groups. Geographical and ethnic factors are known to influence the composition of the gut microbiome, and our results may not fully reflect the variability that exists in a more diverse population. To address this limitation, future studies should include samples from a wider range of geographic regions and ethnic groups. In addition, numerous studies have revealed that there are strong associations between the gut microbiome and CRC, but association does not mean causation. With the accumulation of microbiome dataset, it is necessary to propose statistical methods for assessing causal relationships between the gut microbiome and CRC [45].

Meta-analysis of microbiome studies has always been a hot research topic in recent years, such as in the field of obesity [46], type 2 diabetes [47] and Parkinson’s disease [48]. Large-scale microbiome data can provide a comprehensive and detailed understanding of the microbial communities present in various environments, including the human gut. Meta-analysis studies on the microbiome of more diseases are also worth conducting, because they can help in understanding disease mechanisms and developing intervention treatments. We conducted a meta-analysis on the gut microbiome of CRC patients. However, there is still room for improvement in this study. Incorporating a wide range of microbiome research methods, such as meta-analysis [21], microbiome-based association test analyses [35,49] and microbial differential abundance analyses [37,50], could further enrich our experimental analyses. The dataset used in this study only provides annotation information at the genus level. If the annotated information could be matched to specific sequence information, phylogenetic trees could be constructed to better account for the phylogenetic relationships between microbial communities in the analyses. In addition, exploring metabolite differences between CRC patients and healthy individuals through metabolic pathways in KEGG could provide further insights.

In this study, we systematically analysed the characteristics of gut microbiota in patients with CRC and identified several potential microbial biomarkers. In future studies, we can deepen our understanding of the role of gut microbes in CRC through longitudinal studies, personality medicine and clinical applications and interventions and translate these findings into clinical practice. We can track changes in the gut microbiome over time in CRC patients by conducting longitudinal studies, which in turn provide insights into disease progression and microbiome dynamics during treatment. This will help identify early microbiological signatures that may predict the risk of CRC. In addition, with the spread of precision medicine, we can develop personalized microbiome signatures to predict individual patients’ CRC risk and response to treatment. This will pave the way for tailoring treatment strategies to the characteristics of an individual’s gut microbiome. Future studies could further explore the potential of microbial therapies, such as testing or faecal microbiome transplantation, in preventing and treating CRC and validate the efficacy and safety of these interventions through clinical trials.

## Abbreviations

aMiAD: adaptive microbiome α-diversity-based association analysis.
ANOSIM: analysis of similarity
ASV: amplicon sequence variant
CLR: centred log-ratio
ConQuR: conditional quantile regression
CRC: colorectal cancer
LDA: linear discriminant analysis
LEfSe: linear discriminant analysis effect size
MiRKAT: microbiome regression-based kernel association test
NCBI: National Center for Biotechnology Information
PCA: principal component analysis
PCoA: principal coordinate analysis
PERMANOVA: permutational multivariate analysis of variance
PLSDA: partial least squares discriminant analysis
